# Metabolome-Wide Mendelian Randomization Assessing the Causal Role of Serum and Cerebrospinal Metabolites in Traumatic Brain Injury

**DOI:** 10.3390/biomedicines12061178

**Published:** 2024-05-25

**Authors:** Aojie Duan, Youjia Qiu, Bingyi Song, Yuchen Tao, Menghan Wang, Ziqian Yin, Minjia Xie, Zhouqing Chen, Zhong Wang, Xiaoou Sun

**Affiliations:** Department of Neurosurgery, The First Affiliated Hospital of Soochow University, Suzhou 215006, China; d362922899@163.com (A.D.); qiu_youjia@163.com (Y.Q.);

**Keywords:** traumatic brain injury, serum metabolites, cerebrospinal fluid metabolites, metabolic pathway, Mendelian randomization, causal inference

## Abstract

Previous studies have identified metabolites as biomarkers or potential therapeutic targets for traumatic brain injury (TBI). However, the causal association between them remains unknown. Therefore, we investigated the causal effect of serum metabolites and cerebrospinal fluid (CSF) metabolites on TBI susceptibility through Mendelian randomization (MR). Genetic variants related to metabolites and TBI were extracted from a corresponding genome-wide association study (GWAS). Causal effects were estimated through the inverse variance weighted approach, supplemented by a weighted median, weight mode, and the MR–Egger test. In addition, sensitivity analyses were further performed to evaluate the stability of the MR results, including the MR–Egger intercept, leave-one-out analysis, Cochrane’s Q-test, and the MR-PRESSO global test. Metabolic pathway analysis was applied to uncover the underlying pathways of the significant metabolites in TBI. In blood metabolites, substances such as 4-acetaminophen sulfate and kynurenine showed positive links, whereas beta-hydroxyisovalerate and creatinine exhibited negative correlations. CSF metabolites such as N-formylanthranilic acid were positively related, while kynurenate showed negative associations. The metabolic pathway analysis highlighted the potential biological pathways involved in TBI. Of these 16 serum metabolites, 11 CSF metabolites and metabolic pathways may serve as useful circulating biomarkers in clinical screening and prevention, and may be candidate molecules for the exploration of mechanisms and drug targets.

## 1. Introduction

Traumatic brain injury (TBI) is a neurological condition with a variety of injury mechanisms and tissue pathologies that affect people in all stages of life [1]. It is induced by external mechanical forces that result in brain dysfunction, from mild concussions to severe damage [2]. TBI may cause immediate and delayed brain function alterations. The injury process comprises two stages. The primary injury involves direct brain tissue damage at the moment of impact [3]. Then, in the secondary injury phase, delayed mechanisms such as cerebral edema [4], hypoxia [5], epidural and subdural hematomas, subarachnoid hemorrhage, skull fractures, and diffuse axonal injury further damage brain cells and tissues, exacerbating the injury’s severity [6]. These processing mechanisms underscore the seriousness and complexity of TBI, which requires prompt and comprehensive treatment strategies to deal with immediate and progressive injuries [2].

Recent studies have shown that metabolites are crucial biomarkers for TBI [7,8], and detecting the changes in these metabolites may be a significant tool for evaluating post-TBI neurological recovery and developing therapeutic strategies to minimize the impact of TBI and improve patients’ prognoses [9]. Contemporary studies have primitively elucidated the correlation between TBI and serum metabolite changes. Orešič et al. found an association between TBI severity and specific biomolecules, including two medium-chain fatty acids—decanoic and octanoic acids—and a sugar derivative, 2,3-bisphosphoglyceric acid [7]. Concurrently, Thomas et al. found an inverse association between TBI severity and certain choline phospholipids, including lyso-phosphatidylcholines, ether phosphatidylcholines, and sphingomyelins [10]. However, the specific role and impact of various serum metabolites in TBI patients are not yet fully understood, with studies showing varied and sometimes conflicting results [7,11]. Thus, a deeper investigation considering the controversial role of metabolites that have not been fully identified in TBI is warranted.

Mendelian randomization (MR) is a statistical method for determining causal relationships. MR employs instrumental variables (IVs)—specifically, single-nucleotide polymorphisms (SNPs)—which are strongly linked to exposure to investigate causality between exposures and outcomes, thereby avoiding the effects of confounders and reverse causality. Therefore, using MR analysis for summary statistics taken from genome-wide association studies (GWASs) of metabolite traits could increase the statistical efficacy of causal association [12,13]. MR has been employed to investigate potential causative links between metabolites and neurological disorders such as stroke and Alzheimer’s disease, highlighting the associated connections [14,15,16].

We performed MR estimates to comprehensively explore the causal effects of blood and cerebrospinal fluid (CSF) metabolites on TBI using large-scale GWAS summary data. Moreover, to annotate the biological functions, we further performed metabolic pathway analysis to identify the potential pathways of significant metabolites.

## 2. Materials and Methods

### 2.1. Study Design

**Figure 1:**
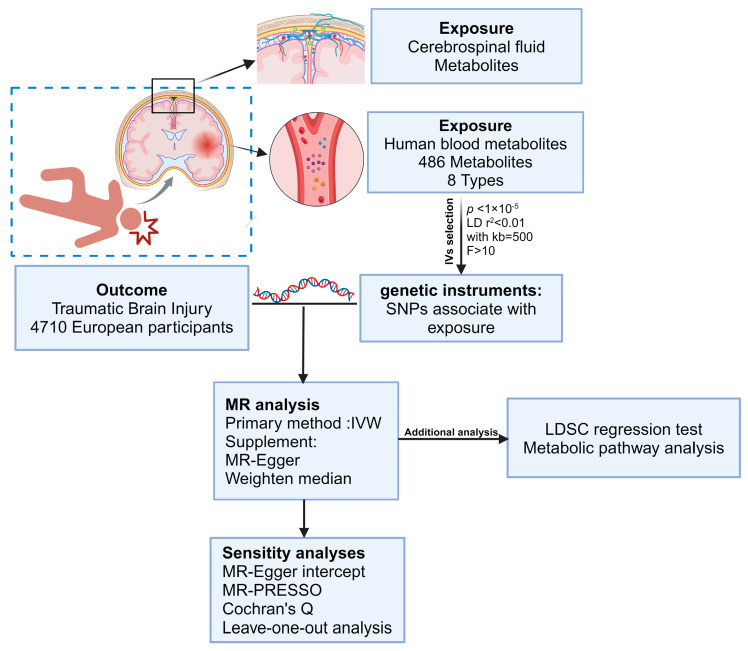
Flow diagram of the study design and data sources showing an overview of the MR analysis design (created with BioRender.com (accessed on 21 January 2024)). IV, instrumental variable; MR, Mendelian randomization; SNPs, single–nucleotide polymorphisms; IVW, inverse–variance weighted; MR–PRESSO, MR pleiotropy residual sum and outlier.

A two-sample MR (TSMR) design was applied to evaluate causal links between serum, CSF metabolites, and TBI. This method should obey three basic assumptions: (1) genetic instruments should be directly associated with metabolites; (2) genetic instruments should not have a connection with the TBI and are not associated with the confounding factors; (3) genetic variants can influence the risk of TBI through metabolites. TBI and metabolite data sets should be obtained from different GWAS summary statistics with no sample overlap. An overview of this MR study is presented in Figure 1.

### 2.2. Source of GWAS Data

Table 1 provides detailed information on the data sources. The serum metabolite GWAS data were sourced from comprehensive and high-quality metabolic analysis research published by Shin et al., which is publicly accessible on the Metabolomics GWAS Server (https://metabolomics.helmholtzmuenchen.de/gwas/ (accessed on 11 December 2023)) [17]. This study enrolled 7824 adult participants from two population cohorts (TwinsUK and KORA), and the associated analysis was performed on more than 21,000 SNPs from the European population. After quality control, 486 metabolites (309 known and 177 unknown) could be used for GWAS analysis (Table S1), including 8 broad metabolic groups: amino acids, carbohydrates, cofactors and vitamins, energy, lipids, nucleotides, peptides, and xenobiotic metabolism [18]. The CSF metabolite GWAS summary data were extracted from a GWAS conducted by Panyard et al. encompassing 338 CSF metabolites in 291 participants (Table S1). These data were also classified into 8 metabolic groups: amino acids, carbohydrates, cofactors and vitamins, energy, lipids, nucleotides, peptides, and xenobiotics [19].

The TBI data were sourced from the latest GWAS meta-analysis published by Kals et al. [20]. After quality control, this study enrolled 4710 European participants from three cohorts (CENTERTBI, TRACKTBI, and MGB). The original research adhered to ethical standards, ensuring that informed consent was acquired from all participants.

### 2.3. Instrumental Variable Identification

To select IVs to signify the connection between an exposure and an outcome, we set different thresholds according to the exposure conditions [21]. Given that genome-wide significance may be too strict for serum metabolites, we extracted IVs from serum metabolites using a relatively relaxed threshold (*p* < 1 × 10^−5^) [22]. Clumping function parameters were defined with a threshold of r^2^ < 0.01 and a minimum distance greater than a 500 kb window, using the 1000 Genomes Project as a reference panel to reduce the potential impact of linkage disequilibrium (LD) on the stochastic allocation of alleles. This method has been widely used in previous MR analysis [23]. Meanwhile, the F statistic was also computed to qualify the strength of IVs through the formula F = beta^2^/se^2^, and IVs with F < 10 were excluded to reduce weak IV bias. We extracted metabolic-associated SNPs from the outcome and discarded SNPs associated with the outcome (*p* < 5 × 10^−5^). Then, we harmonized SNP-exposures and SNP-outcomes and ruled out palindromic SNPs to prevent distortion in strand orientation and allele coding during this process. Finally, we conducted MR analysis on metabolites with more than two SNPs [24].

### 2.4. Statistical Analysis

The inverse variance-weighted (IVW) method was selected as the primary approach to evaluate MR estimates, supplemented by the weighted median and MR–Egger methods [25,26,27]. Odds ratios (ORs) and corresponding 95% confidence intervals (CIs) were applied to present the MR results. The IVW method provides reliable estimates of causal effects, assuming that any genetic variation adheres to our three instrumental variable assumptions and remains unaffected by pleiotropy [25]. The weighted median method allows for the presence of 50% invalid SNPs when combining multiple data on genetic variants into a single causal estimate [26]. With the MR–Egger approach, we evaluated heterogeneity using the presence of intercept terms. When the intercept was constrained to zero, the MR–Egger regression model exhibited similarities to the IVW analyses, indicating the absence of horizontal pleiotropy within the IVs. Therefore, the MR–Egger approach is less precise than the IVW approach in estimating causality. The *p*-value was adjusted based on the false discovery rate (FDR), which adjusts the results of multiple comparisons. A *p* < 0.05 and *q* < 0.1 indicate significant causal association, while *p* < 0.05 and *q* > 0.1 indicate a suggestive causal effect.

Several sensitivity analyses, such as the MR–Egger intercept, Cochran’s Q test, MR-PRESSO, the leave-one-out test, and funnel plots, were undertaken to validate the robustness of the MR estimates. Cochran’s Q test was employed to identify potential heterogeneity, with a *p*-value less than 0.05 indicating the presence of potential heterogeneity. The MR–Egger intercept and MR-PRESSO methods were undertaken to examine the presence of horizontal pleiotropy [28]. Furthermore, we used forest plots and conducted a leave-one-out analysis to explore whether associations were influenced by individual SNP drivers. Therefore, the potential candidate serum metabolites in TBI were determined according to the following conditions: (1) the direction of the three methods was consistent; (2) there was no potential heterogeneity and pleiotropy; (3) there was no SNP with substantial weight assessed in the leave-one-out test.

All statistical analyses were conducted with the R software (version 4.3.0) using the MendelR package and two-sample MR, and MR-PRESSO was performed using the MR-PRESSO package and the LD Score Regression (LDSC) software (version 1.0.1).

### 2.5. Metabolic Pathway Analysis

Metabolic pathway analysis was conducted using MetaboAnalyst 5.0, an online platform accessible at https://www.metaboanalyst.ca/ (accessed on 17 December 2023). Only metabolites that reached statistical significance (*p* < 0.05) were included to ensure the comprehensiveness and reliability of the pathway analysis results. Two databases were employed for this analysis: the Small Molecule Pathway Database (SMPDB) and the Kyoto Encyclopedia of Genes and Genomes (KEGG). The threshold for significance in the pathway analysis was established at 0.10.

### 2.6. Genetic Correlation and Directionality

Prior research indicates that MR estimates may not accurately reflect causal effects given the genetic link between exposure and the outcome of interest, leading to potential false positives [29,30]. Although SNPs associated with TBI were eliminated during IV selection, SNPs without correlations may also mediate the genetics of TBI linkage disequilibrium score (LDSC) regression, which can estimate coinheritance by determining SNP-based chi-square statistics from two traits. Therefore, to explore whether causal effects are not confounded by the correlation between metabolites and TBI, LDSC was employed to investigate that genetic correlation.

## 3. Results

### 3.1. MR Results

Out of 486 metabolites, 3 to 406 independent SNPs were chosen as IVs with an F value exceeding 10, indicating the absence of weak IV bias. The summary information for these SNPs is shown in Table S2.

**Figure 2:**
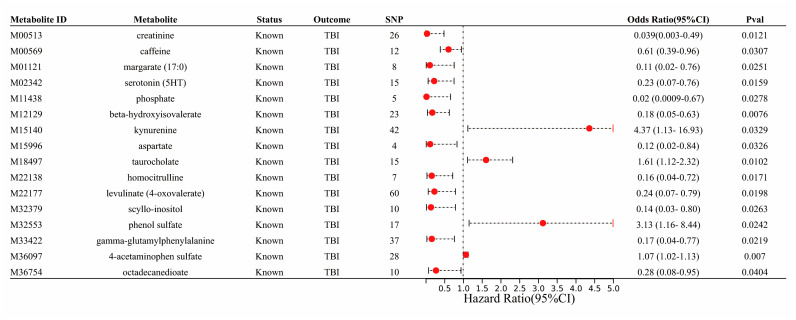
Two–sample MR results between significant serum metabolites and TBI.

Figure 2 and Table S3 show that 26 serum metabolites demonstrated a causal association with TBI risk (*p* < 0.05), including 10 unknown serum metabolites. Among known serum metabolites, 4-acetaminophen sulfate (OR 1.07, 95% CI 1.02 to 1.13, and *p* = 0.007), kynurenine (OR 4.37, 95% CI 1.13 to 16.93, and *p* = 0.03), phenol sulfate (OR 3.13, 95% CI 1.16 to 8.44, and *p* = 0.024), and taurocholate (OR 1.61, 95% CI 1.11 to 2.32, and *p* = 0.012) were positively associated with the risk of TBI, while beta-hydroxyisovalerate (OR 0.18, 95% CI 0.05 to 0.63, and *p* = 0.008), creatinine and serotonin (5HT) (OR 0.23, 95% CI 0.07 to 0.76, and *p* = 0.016), homocitrulline (OR 0.16, 95% CI 0.04 to 0.72, and *p* = 0.017), levulinate (4-oxovalerate) (OR 0.24, 95% CI 0.07 to 0.79, and *p* = 0.02), gamma-glutamylphenylalanine (OR 0.17, 95% CI 0.04 to 0.77, and *p* = 0.022), margarate (17:0) (OR 0.11, 95% CI 0.02 to 0.76, and *p* = 0.025), scylloinositol (OR 0.14; 95% CI 0.03 to 0.80, and *p* = 0.026), phosphate (OR 0.02, 95% CI 0.0009 to 0.67, and *p* = 0.028), caffeine (OR 0.61, 95% CI 0.39 to 0.96, and *p* = 0.03), aspartate (OR 0.12, 95% CI 0.02 to 0.84, *p* = 0.033), and octadecanedioate (OR 0.28, 95% CI 0.08 to 0.95, and *p* = 0.04) were negatively associated with the risk of TBI.

**Figure 3:**
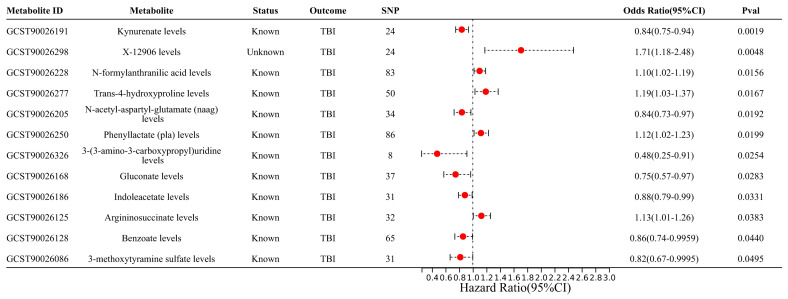
Two–sample MR results between significant CSF metabolites and TBI.

As for CSF metabolites, their IV information is shown in Table S4. Twelve metabolites were identified as having a suggestive causal relationship with the risk of TBI, including one unknown metabolite (Table S5). Of these, N-formylanthranilic acid levels (OR 1.71, 95% CI 1.78 to 2.48, and *p* = 0.005), trans-4-hydroxyproline levels (OR 1.19, 95% CI 1.03 to 1.37, and *p* = 0.017), phenyllactate (pla) levels (OR 1.12, 95% CI 1.02 to 1.23, and *p* = 0.02), and argininosuccinate levels (OR 1.13, 95% CI 1.006 to 1.26, and *p* = 0.038) were positively associated with the risk of TBI, while kynurenate levels (OR 0.84, 95% CI 0.75 to 0.94, and *p* = 0.002), N-acetyl-aspartyl-glutamate (NAAG) levels (OR 0.84, 95% CI 0.73 to 0.97, and *p* = 0.019), 3-(3-amino-3-carboxypropyl)uridine levels (OR 0.48, 95% CI 0.25 to 0.91, and *p* = 0.025), gluconate levels (OR 0.75, 95% CI 0.57 to 0.97, and *p* = 0.028), indoleacetate levels (OR 0.88, 95% CI 0.79 to 0.99, and *p* = 0.033), benzoate levels (OR 0.86, 95% CI 0.74 to 0.996, and *p* = 0.044), and 3-methoxytyramine sulfate levels (OR 0.82, 95% CI 0.67 to 0.9995, and *p* = 0.049) were negatively associated with the risk of TBI (Figure 3). Subsequently, FDR correction was used to identify causal association characteristics. However, the results showed that all metabolites exhibited a suggestive causal relationship with the risk of TBI (*p* < 0.05 and *q* > 0.1).

As IVW methods are susceptible to weak IV bias, sensitivity analysis revealed that the MR–Egger weight median showed the same directions as the MR estimates. Heterogeneity was evaluated using Cochran’s Q statistic in MR–Egger regression. No discernible evidence of notable heterogeneity was found in the effects of instrumental SNPs (Table 2 and Table 3). According to a scatter plot and a funnel plot, the directions of the supplemental methods were consistent with the IVW approach, and IVs were symmetrically distributed, indicating the robustness of the MR estimates (Figures S1–S4, S9 and S10). In addition, the MR–Egger intercept and MR-PRESSO tests did not uncover any horizontal pleiotropy (*p* > 0.05) (Table S6). Moreover, leave-one-out analysis and forest plots did not detect anomalous SNPs (Figures S5–S8, S11 and S12).

### 3.2. Metabolic Pathway Analysis

In the context of known metabolites, we input significant metabolites into Metabolic Analyzer 5.0 to identify the possible metabolic pathways that may participate in TBI pathogenesis. The results of the metabolic pathway analysis are illustrated in Table S7. Kynurenine and serotonin were involved in the metabolic pathways of tryptophan metabolism. Two significant metabolic pathways are related to aspartate: arginine biosynthesis and nicotinate and nicotinamide metabolism. Furthermore, taurocholate takes part in taurine and hypotaurine metabolism, and caffeine is involved in caffeine metabolism. The above metabolites and their related pathways play potential roles in TBI. For CSF metabolites, N-acetyl-aspartyl-glutamate and argininosuccinate were involved in alanine, aspartate, and glutamate metabolism, while N-formylanthranilic acid and indoleacetate were involved in tryptophan metabolism.

### 3.3. Genetic Correlation and Directionality

The LDSC analysis found that the majority of metabolites showed a genetic correlation with TBI, including caffeine (rg = 0.025, se = 0.831, and *p* = 0.976), serotonin (5HT) (rg = 0.847, se = 0.561, and *p* = 0.131), beta-hydroxyisovalerate (rg = −0.644, se = 0.37, and *p* = 0.082), kynurenine (rg = −0.037, se = 0.205, and *p* = 0.856), aspartate (rg = 0.380, se = 0.443, and *p* = 0.391), taurocholate (rg = −0.644, se = 0.370, and *p* = 0.082), levulinate (4-oxovalerate) (rg = −0.317, se = 0.189, and *p* = 0.094), scyllo-inositol (rg = −0.743, se = 0.619, and *p* = 0.230), gamma-glutamylphenylalanine (rg = −0.246, se = 0.412, and *p* = 0.550), and octadecanedioate (rg = −0.089, se = 0.339, and *p* = 0.794). However, due to the small sample size and heritability, we failed to conduct an LDSC analysis for several metabolites (e.g., homocitrulline and phenol sulfate) (Table S8). In the CSF metabolites, five showed a genetic correlation with TBI, including trans-4-hydroxyproline levels (rg = 1.014, se = 1.288, and *p* = 0.431), N-acetyl-aspartyl-glutamate (NAAG) levels (rg = −0.221, se = 0.338, and *p* = 0.514), 3-(3-amino-3-carboxypropyl) uridine levels (rg = −0.068, se = 0.364, and *p* = 0.851), gluconate levels (rg = −0.361, se = 0.590, and *p* = 0.541), and argininosuccinate levels (rg = −0.163, se = 0.286, and *p* = 0.570) (Table S9).

## 4. Discussion

Our MR analysis revealed the causal relationships between serum metabolites, CSF metabolites, and TBI through MR analysis. Overall, 26 critical serum metabolites and 12 CSF metabolites demonstrated causal associations with TBI risk. Of these, 16 serum metabolites and 11 CSF metabolites were currently known. Additionally, ten significant metabolic pathways involved in TBI were detected. Other analyses, such as LDSC and sensitive analysis, also enhanced the credibility of our results. This study represents an initial attempt to methodically evaluate the causal relationship between serum and CSF metabolites in TBI with MR. These findings contribute novel perspectives regarding the impact of gene–environment interactions on the development of TBI and may catalyze future precision medicine.

Recent advances in serum metabolomic studies have identified several serum biomarkers, such as choline phospholipids, that indicate the risk of secondary injury in the aftermath of TBI [10]. In a metabolome-wide study, creatinine was reported to be associated with the severity of TBI [31], and Zheng et al. concluded that elevated creatinine levels are related to a higher frequency of regaining consciousness in male patients [32]. As an efficient scavenger of free radicals, creatinine has demonstrated an antioxidant effect on the brain that decreases oxidative stress in the secondary injury response of TBI [33]. Rangasamy et al. concluded that oral medication with sodium benzoate could reduce vascular damage and decrease the size of lesion cavities in a mouse model of TBI. Furthermore, an improvement in memory and motor function was also observed in the mice, indicating the therapeutic potential of benzoate in TBI [34]. In another study, a positive causal relationship was identified between phenyllactate and TBI, with previous evidence showing that phenyllactate could induce reactive oxygen species formation and lead to DNA oxidative damage in glial cells [35].

The connection between TBI and hyperglycemia is notably significant. Bosarge et al. underscored that stress-induced hyperglycemia was not only related to increased mortality and adverse prognosis in TBI individuals, but also induced underlying metabolic disruptions [36]. These metabolic disruptions are likely a result of acute sympathetic responses and compromised brain metabolism, leading to an increased glucose demand. Pappacena et al. also found that greater dysglycemia correlates with higher mortality rates among TBI patients [37]. Therefore, implementing effective glucose management strategies is crucial for reducing the severity of TBI and enhancing patient recovery. In our study, gluconate on the CSF level was inversely associated with TBI, and it might be a promising biomarker for predicting TBI. An observational study also noted a decrease in gluconate in TBI patients [38]. Additionally, gluconate has antioxidative properties, which may also play a role in decreasing oxidative stress [39].

Our metabolic pathway analysis also identified metabolites from human blood and CSF metabolites involved in tryptophan metabolism through three main pathways: indole, serotonin, and kynurenine. Although the role of indoleacetate in TBI was not detected, the indole pathway of tryptophan metabolism changed at seven days post-TBI, which is related to the progress of TBI [40]. Moreover, kynurenine and serotonin play pivotal roles in the tryptophan metabolism pathway. The kynurenine pathway of tryptophan metabolism generates various neuroactive metabolites [41]. Dysregulation in this pathway may serve as a potential molecular mechanism through which TBI triggers a persistent neuroinflammatory response [41]. Zhang et al. concluded that kynurenine levels were markedly elevated 21 days post-TBI, while the ratios of serotonin to tryptophan and melatonin to tryptophan exhibited significant decreases at the same time point [42]. Kynurenate levels, also known as kynurenic acids (KYNAs) [19], are a downstream product of kynurenine created through a kynurenic pathway that exhibits a neuroprotective effect in TBI [43]. Our MR estimates identified a negative causal relationship between them. KYNAs are a non-competitive antagonist for α7 nicotinic acetylcholine receptors (α7nAChRs) that exert anti-inflammatory effects [44], and Kelso et al. concluded that α7nAChRs are associated with the excitotoxicity and severity of TBI [45]. Therefore, KYNAs may exhibit inflammatory effects in the pathogenesis of TBI. Additionally, 3-methoxytyramine is a dopaminergic metabolite that accentuates the intricate relationship between neurotransmitter dynamics and tryptophan metabolism [41,46]. N-formylanthranilic acid is a benzoic biosynthesized from N-formylkynurenine from kynureninase [46]. Our analysis identified a positive causal association between N-formylanthranilic acid and TBI, which may be attributable to inflammation activation [46].

N-acetyl-aspartyl-glutamate (NAAG) has demonstrated a neuroprotective influence on TBI [46,47], and it also exhibited a protective effect in our MR analysis. TBI sets off a multifaceted series of biochemical and physiological reactions, leading to significant alterations in the release and metabolism of neurotransmitters, with NAAG being particularly noteworthy [48]. Research suggests that intervention with NAAG modulates synaptic transmission, mainly by inhibiting the release of glutamate, a primary neurotoxicity mechanism in TBI [47,49]. MR estimates have found that the NAAG peptidase inhibitor increases NAAG levels in the dorsal hippocampus after TBI in mice while also reducing levels of glutamate, aspartate, and GABA [50]. Previous studies have advised that post-TBI alterations in the levels of NAAG are intricately related to the severity of subsequent injuries, encompassing neuroinflammation, cellular apoptosis, and neurodegeneration [47]. However, despite these insights, our current understanding of NAAG’s potential as a therapeutic intervention remains limited, and further research is required to substantiate these preliminary findings and explore their clinical applications in treating TBI.

Caffeine is the most widely used psychostimulant. Studies have found that it has a neuroprotective effect on neurodegenerative diseases. Our MR found that it was negatively associated with TBI, indicating its protective potential. In a multicenter study, low-to-median caffeine levels were connected to more significant functional recovery and survival after TBI [51]. Furthermore, earlier research demonstrated that chronic caffeine consumption before injury ameliorates neurological deficits, reduces brain swelling, and decreases infiltration by inflammatory cells in mice [52]. Trans-4-hydroxyproline is reportedly the therapeutic target of traditional Chinese medicine in TBI [53]. Aspartate is involved in arginine metabolism and is negatively associated with TBI in MR estimates; Menshchikov et al. also observed decreased aspartate levels after mild TBI [54]. Taurocholate is reported to be positively associated with TBI and involved in taurine and hypotaurine metabolism. Zheng et al. observed significant pathological profiles for arginine, taurine, and hypotaurine metabolisms in the hippocampus of acute TBI [55]. Wen et al. also concluded that taurine and hypotaurine metabolisms could protect neurons from the influence of inflammation and relate to hypoxic preconditioning [56].

Our MR analysis has several advantages. First, ours is the first study to date to entirely and systematically analyze the causal influence of serum metabolites and CSF metabolites on TBI. The positive metabolites we discovered may become promising biomarkers for TBI, contributing to clinical risk prediction and routine screening. Second, we ruled out reverse causality and confounding interference possibilities through rigorous MR analysis. Rigorous outcome inclusion metrics and sensitivity analyses also confirmed the robustness of our MR results. Third, we assessed the heritability of IVs using LDSC and metabolic pathway analysis, which made the MR estimates more robust. However, some limitations should also be noted. First, although there is a comprehensive spectrum of metabolites in blood and CSF, the function and underlying mechanisms of these metabolites in TBI were not fully elucidated in our study, which constrained the interpretation of our MR estimates. Second, the metabolite GWAS data covered a wide range of diseases and ages without gender and age stratification. Third, our data source is limited to a European population and, as such, a further cross-ethnic survey is required. Prospective cohort studies across multiple centers would be beneficial in exploring and validating the association between significant metabolites and TBI. Finally, our results demonstrated the causal relationship between related metabolites and TBI in MR analysis, and we did not further investigate potential mechanisms and therapeutic potential through different perspectives. Targeting metabolomic changes in TBI patients and changes in specific metabolic pathways should be the direction of future research. In addition, multi-perspective validations, such as metabolomic measurement and single-cell RNA sequencing [57], are required to identify changes in metabolic traits at the cellular level, which brings benefit to identifying underlying mechanisms between metabolic changes and TBI and provides theoretical support for clinical practice. Overall, we need to further explore the exact association between these positive metabolites and specific diseases or physiological processes, as well as their potential roles in treatment. Such research goes beyond chemical properties and includes a deeper understanding of biological mechanisms, such as related signaling pathways, protein interactions, etc.

## 5. Conclusions

In conclusion, this is the first MR study to comprehensively evaluate the causal relationship between serum metabolites, CSF metabolites, and the risk of TBI. Overall, 16 serum metabolites and 11 known CSF metabolites were identified as having suggestive causal associations with TBI. A metabolic pathway analysis found ten related pathways that played a role in the pathogenesis of TBI, which may help us to better comprehend the underlying mechanisms of TBI. These metabolites may serve as useful circulating biomarkers in clinical screening and prevention, and could also be candidate molecules for the exploration of mechanisms and drug targets.

## Figures and Tables

**Table 1:** Data sources for used GWAS summary statistics.

Characteristic	Resource	Sample Size	Year	Population
Exposure				
Serum metabolites	Shin et al. [17]	7824	2017	European
CSF ^1^ metabolites	Panyard et al. [19]	291	2017	European
Outcome				
TBI ^2^	Kals et al. [20]	4710	2023	European

^1^ CSF: cerebrospinal fluid; ^2^ TBI: traumatic brain injury.

**Table 2:** Primary MR results and sensitivity analyses for causality of serum metabolites in TBI.

Serum Metabolites	Method	SNP	Pval	OR (95% CI)	Cochran Q Test	Pleiotropy Test	MR-PRESSO Global Test
creatinine	IVW ^1^	26	0.012	0.039 (0.0031, 0.4915)	0.827	0.313	0.831
caffeine	IVW ^1^	12	0.031	0.6122 (0.3924, 0.9553)	0.508	0.466	0.516
kynurenine	IVW ^1^	42	0.033	4.368 (1.1272, 16.9261)	0.179	0.258	0.184
aspartate	IVW ^1^	4	0.033	0.1207 (0.0174, 0.8391)	0.486	0.313	0.535
taurocholate	IVW ^1^	15	0.010	1.6133 (1.1199, 2.3243)	0.819	0.253	0.856
homocitrulline	IVW ^1^	7	0.017	0.1636 (0.0369, 0.7242)	0.367	0.476	0.412
levulinate (4-oxovalerate)	IVW ^1^	60	0.020	0.236 (0.0701, 0.7948)	0.297	0.336	0.31
scyllo-inositol	IVW ^1^	10	0.026	0.1431 (0.0258, 0.7951)	0.879	0.382	0.901
phenol sulfate	IVW ^1^	17	0.024	3.1293 (1.1601, 8.4411)	0.445	0.562	0.465
gamma-glutamylphenylalanine	IVW ^1^	37	0.022	0.166 (0.0357, 0.7709)	0.886	0.863	0.904
4-acetaminophen sulfate	IVW ^1^	28	0.007	1.0748 (1.0199, 1.1326)	0.256	0.179	0.011
octadecanedioate	IVW ^1^	10	0.040	0.2778 (0.0816, 0.9457)	0.160	0.767	0.03
margarate (17:0)	IVW ^1^	8	0.025	0.1136 (0.0169, 0.7623)	0.543	0.024	0.49
serotonin (5HT)	IVW ^1^	15	0.016	0.229 (0.0691, 0.7589)	0.656	0.178	0.658
phosphate	IVW ^1^	5	0.028	0.0246 (0.0009, 0.6679)	0.950	0.637	0.975
beta-hydroxyisovalerate	IVW ^1^	23	0.008	0.1792 (0.0507, 0.633)	0.523	0.862	0.603

^1^ IVW: inverse variance-weighted.

**Table 3:** Primary MR results and sensitivity analyses for causality of CSF metabolites in TBI.

Metabolites	Method	SNP	Pval	OR	Cochran Q Test	Pleiotropy Test	MR-PRESSO Global Test
Indoleacetate levels	IVW ^1^	31	0.049457	0.8162 (0.6665, 0.9995)	0.651	0.309	0.645
Argininosuccinate levels	IVW ^1^	32	0.038322	1.1256 (1.0064, 1.2589)	0.682	0.601	0.694
Benzoate levels	IVW ^1^	65	0.044046	0.8562 (0.7361, 0.9959)	0.146	0.644	0.156
Gluconate levels	IVW ^1^	37	0.028267	0.7464 (0.5747, 0.9693)	0.199	0.551	0.219
Indoleacetate levels	IVW ^1^	31	0.033053	0.8836 (0.7885, 0.9901)	0.651	0.309	0.645
Kynurenate levels	IVW ^1^	24	0.001944	0.8377 (0.7489, 0.937)	0.542	0.847	0.588
N-acetyl-aspartyl-glutamate (NAAG) levels	IVW ^1^	34	0.019227	0.8398 (0.7255, 0.972)	0.945	0.869	0.949
N-formylanthranilic acid levels	IVW ^1^	83	0.015553	1.0998 (1.0182, 1.1879)	0.481	0.572	0.496
Phenyllactate (pla) levels	IVW ^1^	86	0.01988	1.1192 (1.018, 1.2305)	0.577	0.061	0.584
Trans-4-hydroxyproline levels	IVW ^1^	50	0.016672	1.1904 (1.0321, 1.3729)	0.931	0.041	0.934
X-12906 levels	IVW ^1^	24	0.004793	1.7082 (1.1775, 2.4781)	0.588	0.156	0.631
3-(3-amino-3-carboxypropyl)uridine levels	IVW ^1^	8	0.025419	0.4764 (0.2486, 0.9128)	0.664	0.922	0.696

^1^ IVW: inverse variance-weighted.

## Data Availability

All data used in this study are publicly available from the cited sources.

## Supplementary Materials

The following supporting information can be downloaded at https://www.mdpi.com/article/10.3390/biomedicines12061178/s1: Figure S1: Scatter plot of MR results for known serum metabolites and TBI. Figure S2: Scatter plot of MR results for unknown serum metabolites and TBI. Figure S3: Funnel plot of MR results for known serum metabolites and TBI. Figure S4: Funnel plot of MR results for unknown serum metabolites and TBI. Figure S5: Leave-one-out plot of MR results for known serum metabolites and TBI. Figure S6: Leave-one-out plot of MR results for unknown serum metabolites and TBI. Figure S7: Forest plot of MR results for known serum metabolites and TBI. Figure S8: Forest plot of MR results for unknown serum metabolites and TBI. Figure S9: Scatter plot of MR results for CSF metabolites and TBI. Figure S10: Funnel plot of MR results for cerebrospinal fluid metabolites and TBI. Figure S11: Leave-one-out plot of MR results for cerebrospinal fluid metabolic and TBI. Figure S12: Forest plot of MR results for cerebrospinal fluid metabolites and TBI. Table S1: Identification (ID) list for each blood and cerebrospinal fluid metabolite. Table S2: Instrumental variants identified in serum metabolites. Table S3: MR results for 486 serum metabolites in TBI. Table S4: Instrumental variants identified in CSF metabolites. Table S5: MR results for 338 CSF metabolites in TBI. Table S6: Heterogeneity and MR-PRESSO test results. Table S7: Metabolic pathways with significant serum and CSF metabolite enrichment. Table S8: SNP-based heritability (h2) of the metabolites. Table S9: Rg results.
