# Supplementary material for: Metabolome-Wide Mendelian Randomization Assessing the Causal Role of Serum and Cerebrospinal Metabolites in Traumatic Brain Injury
Source: Biomedicines. 2024 May 25;12(6):1178. doi: 10.3390/biomedicines12061178 (PMC11201266; doi:10.3390/biomedicines12061178)
Supplement: Supplementary file 1 [file biomedicines-12-01178-s001.zip › supplementary figures.pdf]

# Metabolome-Wide Mendelian Randomization Assessing the Causal Role of Human Blood and Cerebrospinal Metabolites in Traumatic Brain Injury

**Figure legends**

Figure S1. Forest plot of association of genetically proxied drug targets with risk of coronary heart Scatter plot for MR result of the known serum metabolites and TBI;

Figure S2: Scatter plot for MR result of the unknown serum metabolites and TBI;

Figure S3: Funnel plot for MR result of the known serum metabolites and TBI;

Figure S4: Funnel plot for MR result of the unknown serum metabolites and TBI;

Figure S5: Leave one out for MR result of the known serum metabolites and TBI;

Figure S6: Leave one out for MR result of the unknown serum metabolites and TBI;

Figure S7: forest for MR result of the known serum metabolites and TBI;

Figure S8: forest for MR result of the unknown serum metabolites and TBI;

Figure S9: Scatter plot for MR result of the CSF metabolites and TBI;

Figure S10: Funnel plot for MR result of the cerebrospinal fluid metabolites and TBI;

Figure S11: Leave-one-out plot for MR result of the cerebrospinal fluid metabolic and TBI;

Figure S12: Forest plot for MR result of the cerebrospinal fluid metabolites and TBI.

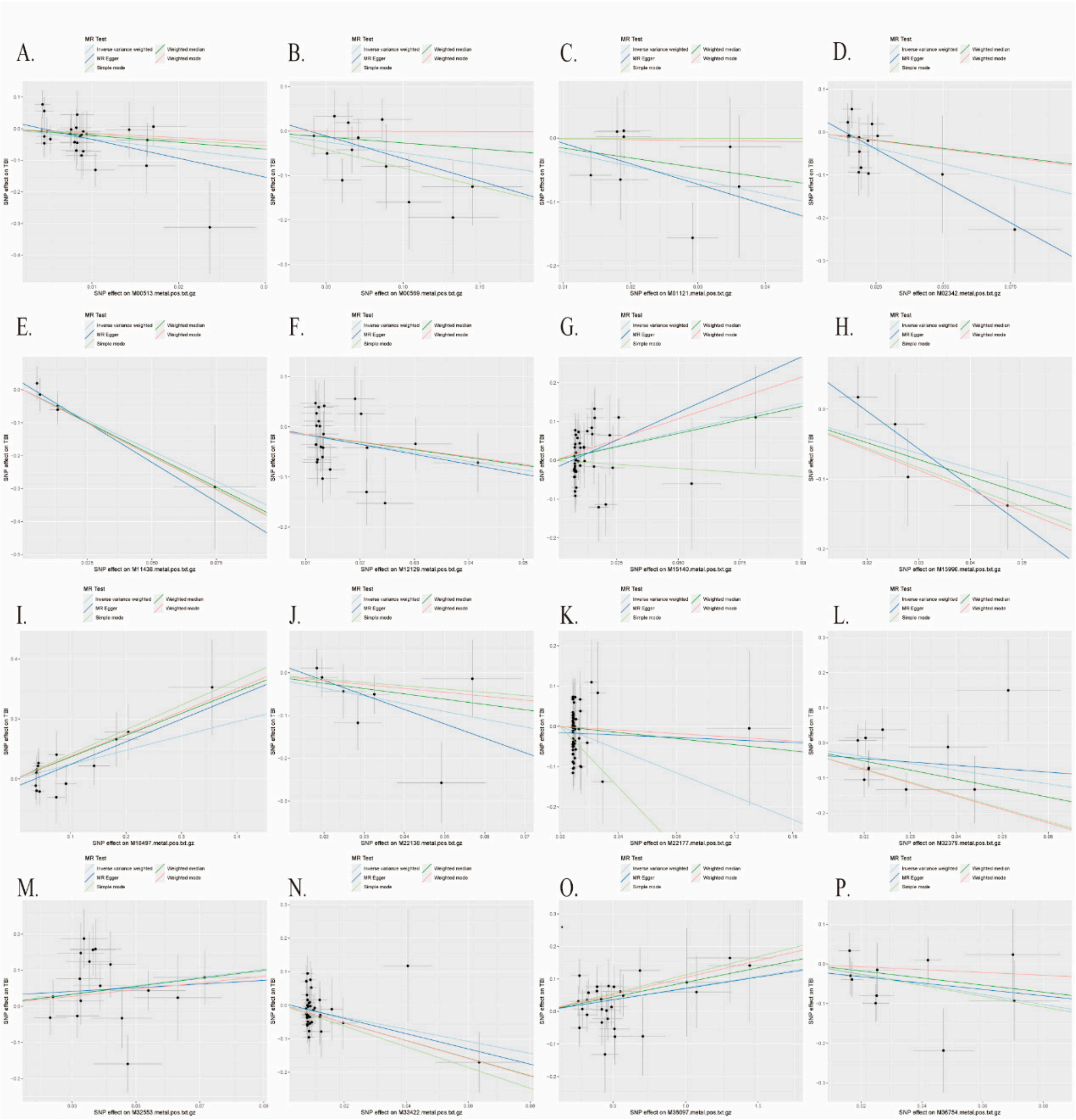


Figure S1. Scatter plot for MR result of the known serum metabolites and TBI. (A) Creatinine; (B) Caffeine; (C) Margarate (17:0); (D) Serotonin (5HT); (E) Phosphate; (F) Beta-hydroxyisovalerate; (G) Kynurenine; (H) Aspartate; (I) Taurocholate; (J) Homocitrulline; (K) Levulinate (4-oxovalerate); (L) Scyllo-inositol; (M) Phenol sulfate; (N) Gamma-glutamylphenylalanine; (O) 4-acetaminophen sulfate; (P) Octadecanedioate;

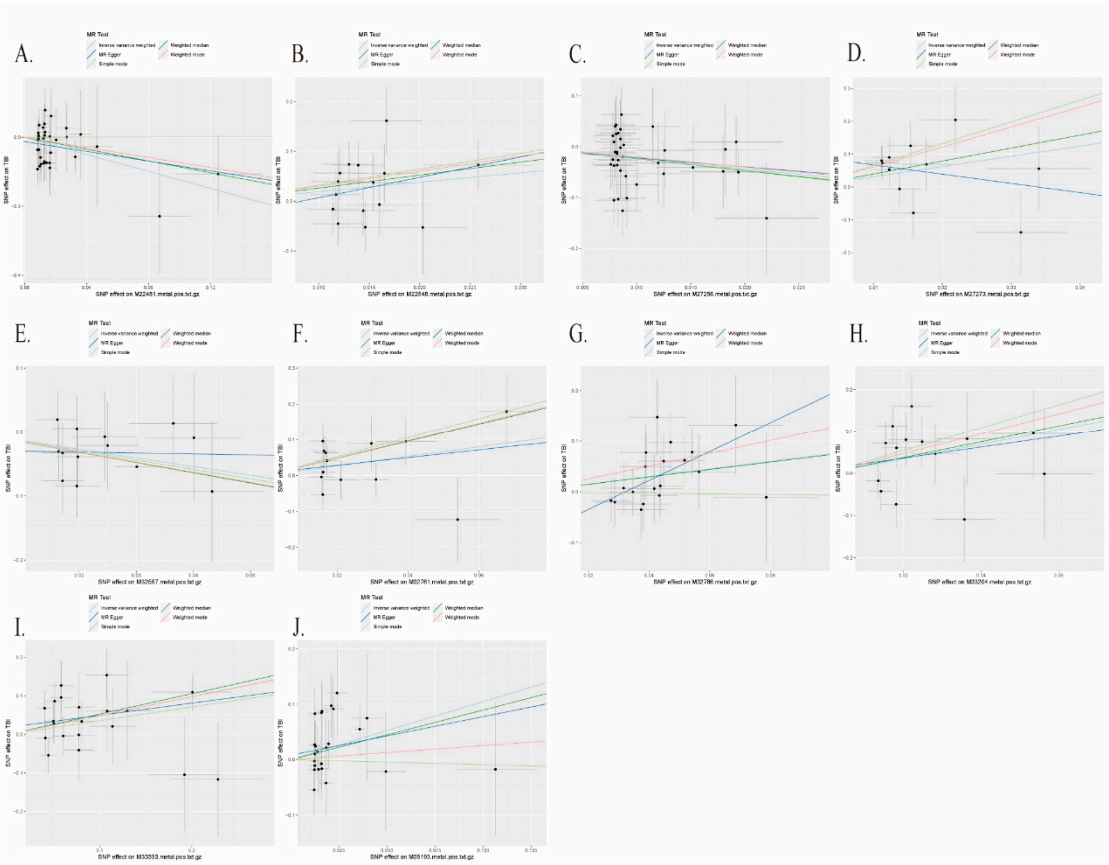


Figure S2. Scatter plot for MR result of the unknown serum metabolites and TBI.
(A) X-08988; (B) X-09026; (C) X-10500; (D) X-10506; (E) X-02249; (F) X-11444; (G) X-11469; (H) X-11859; (I) X-12007; (J) X-13435;

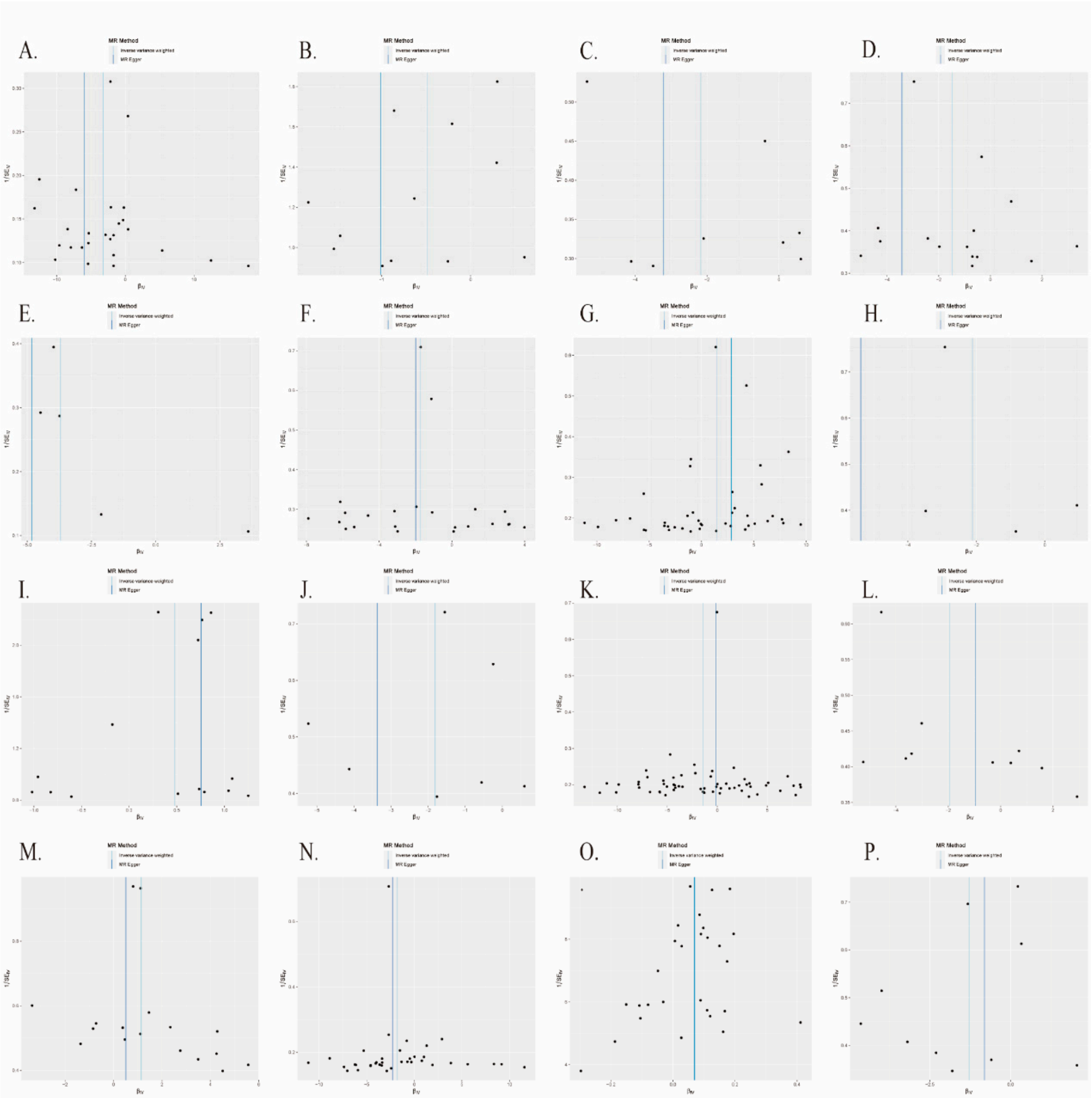


Figure S3. Funnel plot for MR result of the known serum metabolites and TBI.
(A) Creatinine; (B) Caffeine; (C) Margarate (17:0); (D) Serotonin (5HT); (E) Phosphate; (F) Beta-hydroxyisovalerate; (G) Kynurenine; (H) Aspartate; (I) Taurocholate; (J) Homocitrulline; (K) Levulinate (4-oxovalerate); (L) Scyllo-inositol; (M) Phenol sulfate; (N) Gamma-glutamylphenylalanine; (O) 4-acetaminophen sulfate; (P) Octadecanedioate;

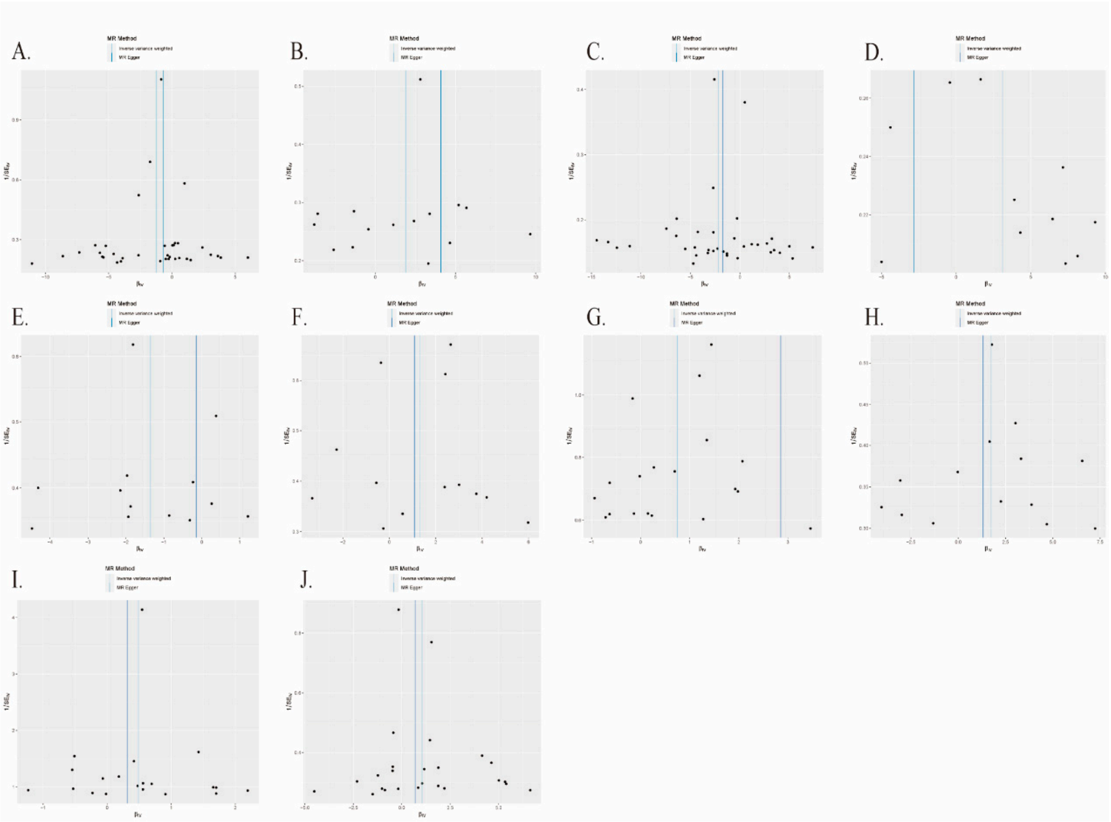


Figure S4. Funnel plot for MR result of the unknown serum metabolites and TBI.
(A) X-08988; (B) X-09026; (C) X-10500; (D) X-10506; (E) X-02249; (F) X-11444; (G) X-11469; (H) X-11859; (I) X-12007; (J) X-13435;

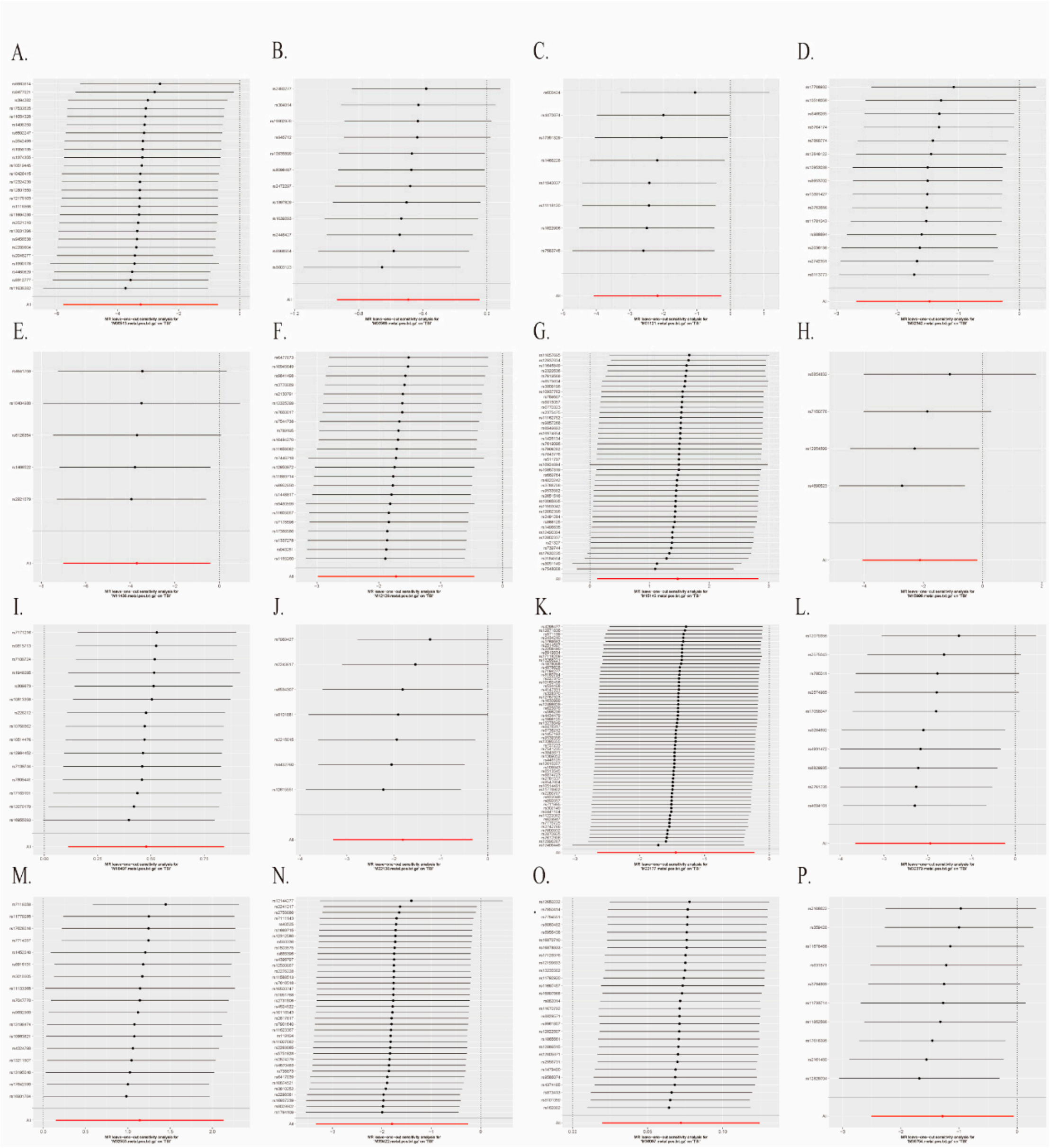


Figure S5. Leave one out for MR result of the known serum metabolites and TBI. (A) Creatinine; (B) Caffeine; (C) Margarate (17:0); (D) Serotonin (5HT); (E) Phosphate; (F) Beta-hydroxyisovalerate; (G) Kynurenine; (H) Aspartate; (I) Taurocholate; (J) Homocitrulline; (K) Levulinate (4-oxovalerate); (L) Scyllo-inositol; (M) Phenol sulfate; (N) Gamma-glutamylphenylalanine; (O) 4-acetaminophen sulfate; (P) Octadecanedioate;

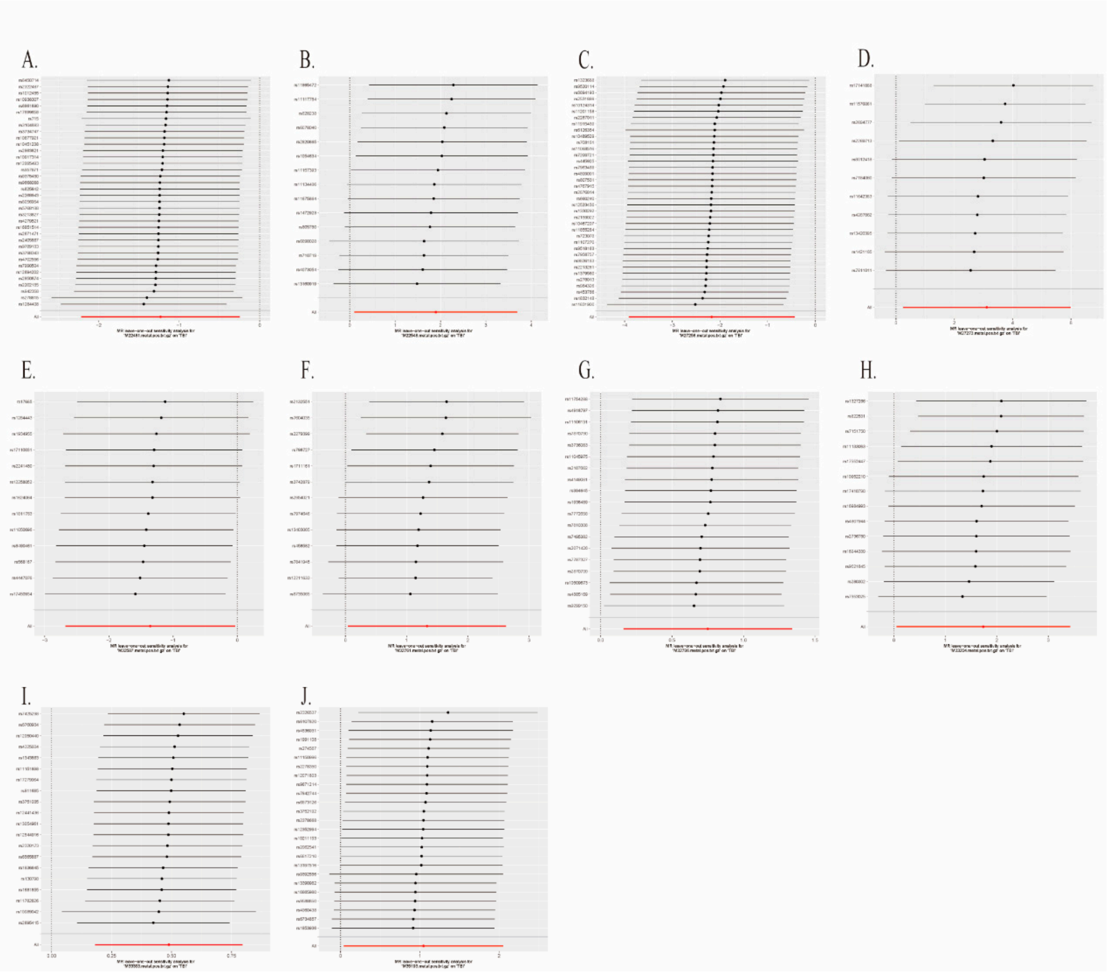


Figure S6. Leave one out for MR result of the unknown serum metabolites and TBI. (A) X-08988; (B) X-09026; (C) X-10500; (D) X-10506; (E) X-02249; (F) X-11444; (G) X-11469; (H) X-11859; (I) X-12007; (J) X-13435;

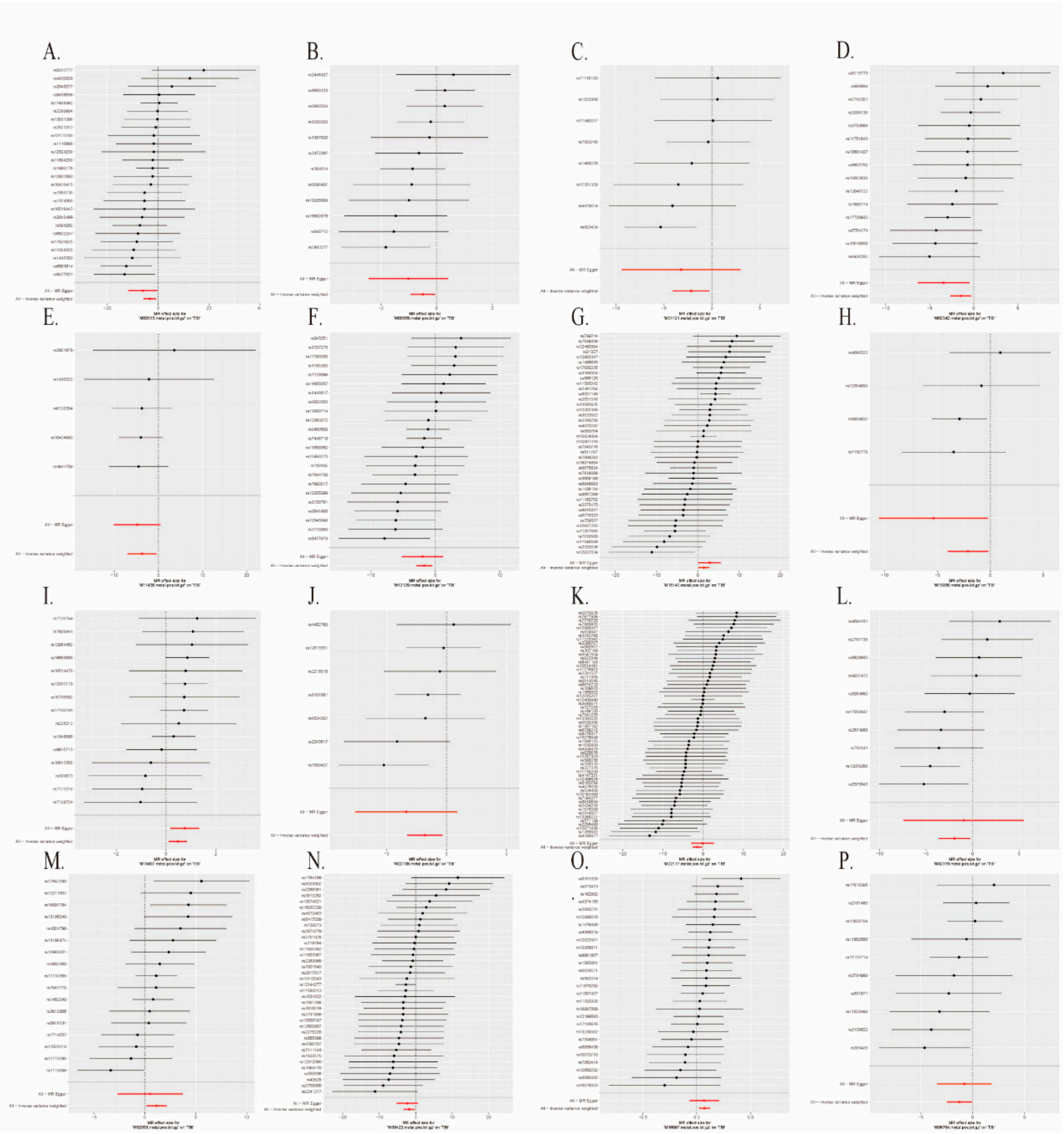


FigureS7. forest for MR result of the known serum metabolites and TBI.
(A) Creatinine; (B) Caffeine; (C) Margarate (17:0); (D) Serotonin (5HT); (E) Phosphate; (F) Beta-hydroxyisovalerate; (G) Kynurenine; (H) Aspartate; (I) Taurocholate; (J) Homocitrulline; (K) Levulinate (4-oxovalerate); (L) Scyllo-inositol; (M) Phenol sulfate; (N) Gamma-glutamylphenylalanine; (O) 4-acetaminophen sulfate; (P) Octadecanedioate;

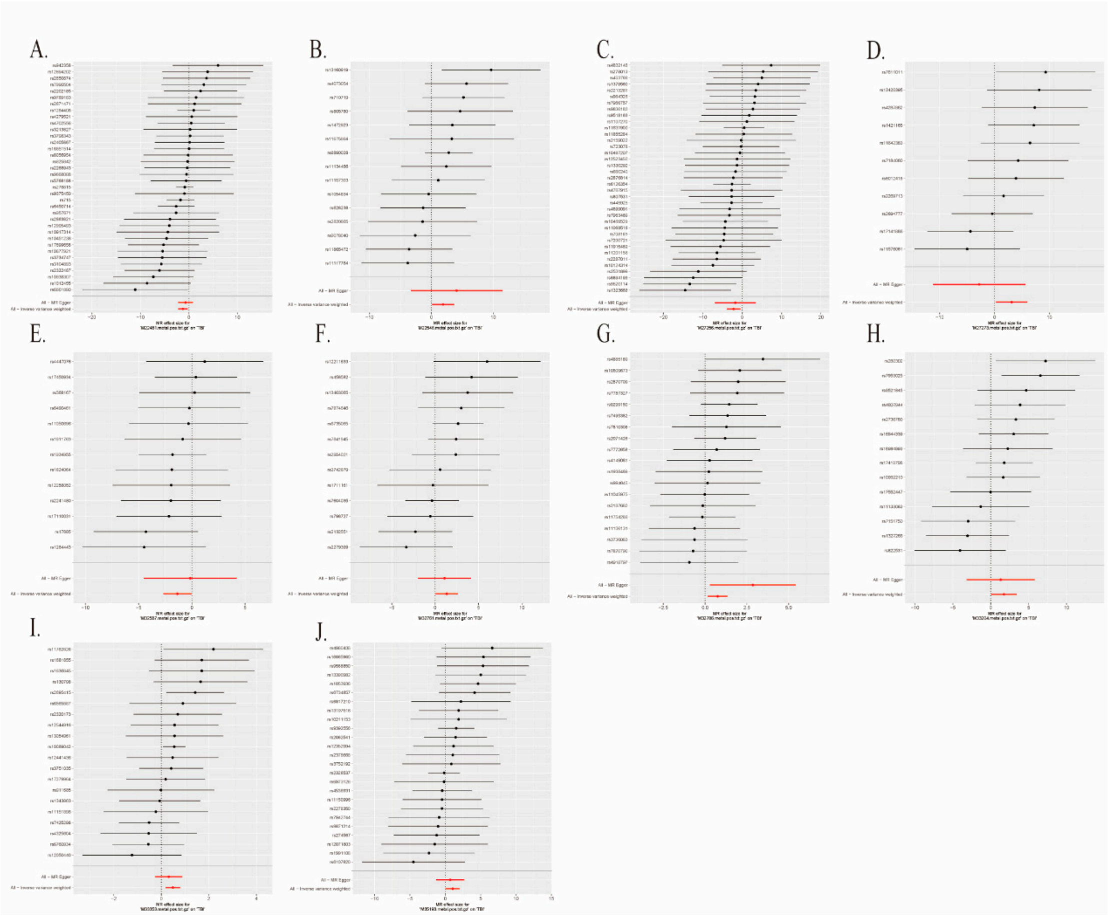

FigureS8. forest for MR result of the unknown serum metabolites and TBI.
(A) X-08988; (B) X-09026; (C) X-10500; (D) X-10506; (E) X-02249; (F) X-11444; (G) X-11469; (H) X-11859; (I) X-12007; (J) X-13435;

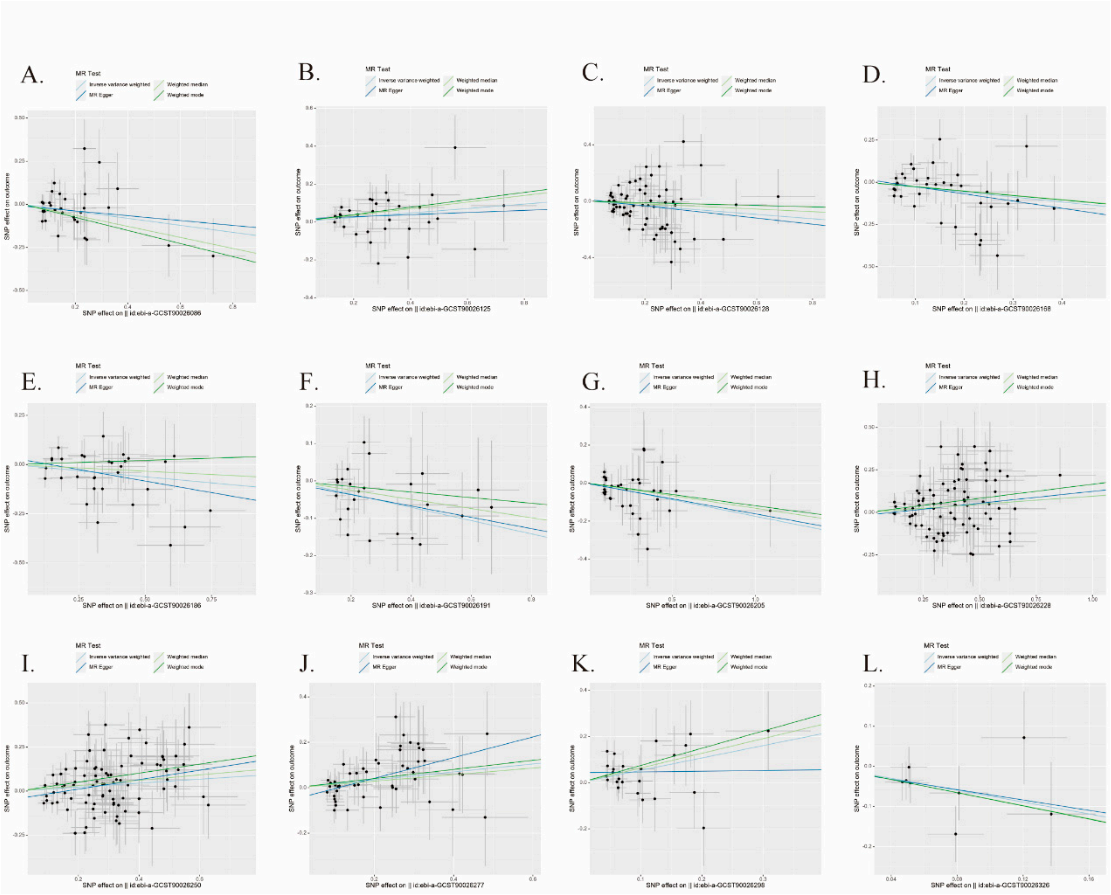


Figure S9. Scatter plot for MR result of the cerebrospinal fluid metabolites and TBI. (A) 3-methoxytyramine sulfate levels; (B) Argininosuccinate levels; (C) Benzoate levels; (D) Gluconate levels; (E) Indoleacetate levels; (F) Kynurenate levels; (G) N-acetyl-aspartyl-glutamate (naag) levels; (H) N-formylanthranilic acid levels; (I) Phenyllactate (pla) levels; (J) Trans-4-hydroxyproline levels; (K) X-12906 levels; (L) 3-(3-amino-3-carboxypropyl)uridine levels;

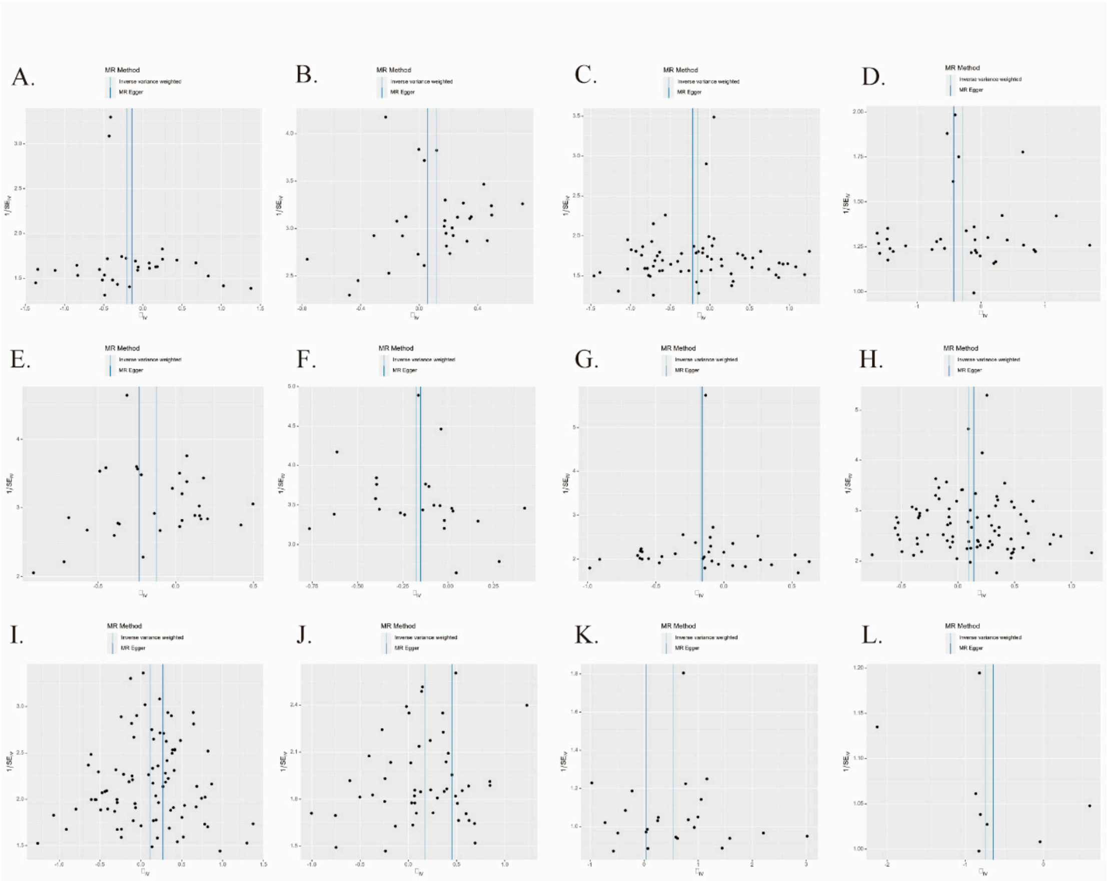


Figure S10. Funnel plot for MR result of the cerebrospinal fluid metabolites and TBI. (A) 3-methoxytyramine sulfate levels; (B) Argininosuccinate levels; (C) Benzoate levels; (D) Gluconate levels; (E) Indoleacetate levels; (F) Kynurenate levels; (G) N-acetyl-aspartyl-glutamate (naag) levels; (H) N-formylanthranilic acid levels; (I) Phenyllactate (pla) levels; (J) Trans-4-hydroxyproline levels; (K) X-12906 levels; (L) 3-(3-amino-3-carboxypropyl)uridine levels;

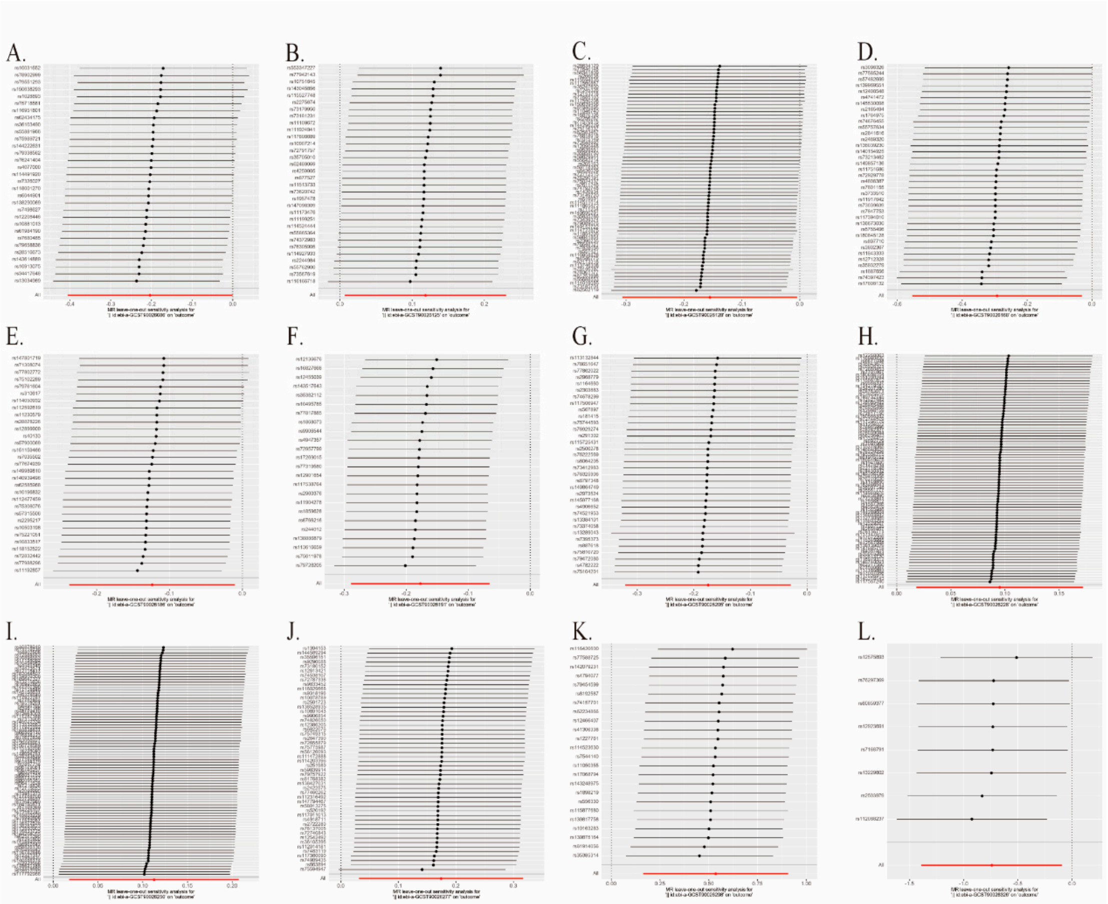


Figure S11. Leave-one-out plot for MR result of the cerebrospinal fluid metabolic and TBI.

(A) 3-methoxytyramine sulfate levels; (B) Argininosuccinate levels; (C) Benzoate levels; (D) Gluconate levels; (E) Indoleacetate levels; (F) Kynurenate levels; (G) N-acetyl-aspartyl-glutamate (naag) levels; (H) N-formylanthranilic acid levels; (I) Phenyllactate (pla) levels; (J) Trans-4-hydroxyproline levels; (K) X-12906 levels; (L) 3-(3-amino-3-carboxypropyl)uridine levels;

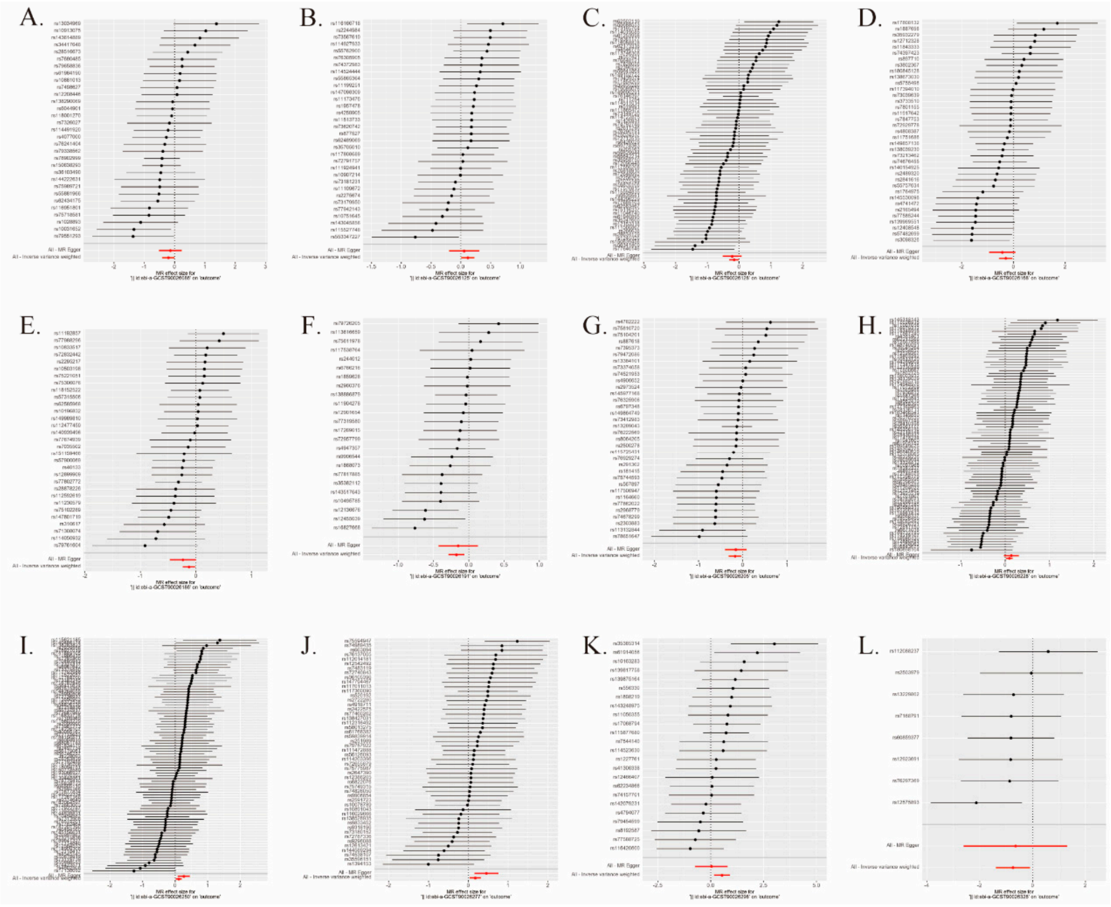


Figure S12. Forest plot for MR result of the cerebrospinal fluid metabolites and TBI. (A) 3-methoxytyramine sulfate levels; (B) Argininosuccinate levels; (C) Benzoate levels; (D) Gluconate levels; (E) Indoleacetate levels; (F) Kynurenate levels; (G) N-acetyl-aspartyl-glutamate (naag) levels; (H) N-formylanthranilic acid levels; (I) Phenyllactate (pla) levels; (J) Trans-4-hydroxyproline levels; (K) X-12906 levels; (L) 3-(3-amino-3-carboxypropyl)uridine levels;
